# Identification of the important environmental factors influencing natural vegetation succession following cropland abandonment on the Loess Plateau, China

**DOI:** 10.7717/peerj.10349

**Published:** 2020-11-13

**Authors:** Zhenguo Zhang, Mingming Wang, Jikai Liu, Xinwei Li

**Affiliations:** 1 College of Resources and Environment, Anhui Science and Technology University, Fengyang, Anhui, China; 2 Northeast Institute of Geography and Agroecology, Chinese Academy of Sciences, Changchun, Jilin, China; 3 Key Laboratory of Mollisols Agroecology, Chinese Academy of Sciences, Changchun, Jilin, China

**Keywords:** Vegetation succession, C/N ratio, Abandoned cropland, Loess Plateau, Classification tree model

## Abstract

Identification of typical vegetation succession types and their important influencing factors is an important prerequisite to implement differential vegetation and soil management after land abandonment on the Loess Plateau, China. However, there is no reported study specifically on the identification of vegetation types and their important factors as well as the thresholds of the important factors for classification of the vegetation types, based on the medium- to long-term succession of natural vegetation after cropland abandonment. We collected vegetation and soil data on the natural vegetation with the longest 60-year-old forest communities that developed after cropland abandonment and analyzed the data using two-way indicator species analysis, detrended correspondence analysis, direct canonical correspondence analysis and classification tree model. The vegetation communities were classified into five distinct vegetation types, including *Artemisia scoparia*, *Lespedeza davurica* and *Stipa bungeana*, *Artemisia giraldii pamp*, *Sophora viciifolia*, *Quercus liaotungensis* and *Biota orientalis*. The years after cropland abandonment and soil C/N were further identified as important factors determining the types of vegetation. Likewise, it was observed that most of the investigated soil nutrient variables and soil texture-related variables improved with the vegetation succession while soil water in the surface layers showed a decreasing trend. These findings may provide an ecological basis for site-specific management of vegetation types after cropland abandonment in the medium-long term on the Loess Plateau. Our results encourage further exploration of vegetation succession and their important factors based on longer periods of vegetation succession after cropland abandonment under more soil and climatic conditions on the mountainous areas as the Loess Plateau.

## Introduction

Soil erosion is a significant cause of soil degradation, productivity decline, and ecological deterioration (Geist & Lambin, 2004; Fu et al., 2011; Zhu et al., 2019). Erosion is caused by various mechanisms, including biophysical factors with socio-economic, technological, demographic, political, and cultural influences (Geist & Lambin, 2004; Fu et al., 2011). Vegetation is a biophysical factor that impedes soil erosion by protecting the soil surface from rainfall and improving soil quality with the development of vegetation communities (Cerdà, 1997; Bullock et al., 2011; Deng et al., 2018). Vegetation succession refers to the process of restoring plant communities and improving soil quality (Walker, Walker & Hobbs, 2007; Zhao et al., 2019; Peña Angulo et al., 2019; Lasanta, Arnáez & Nadal-Romeroa, 2019).

Recently, the rate and extent of global cropland abandonment has increased greatly since the 1950s due to rural depopulation, market incentives, and ecological restoration programs (Campbell et al., 2008; Cramera, Hobbs & Standisha, 2008; Zhang et al., 2013; Zhang et al., 2018). Consequently, greater attention has been paid to the process of vegetation succession and its impact on soil quality on abandoned land, especially in mountainous areas (Jia et al., 2011; Lasanta, Arnáez & Nadal-Romeroa, 2019; Romero, Pereira & Martínez-Murillo, 2019). The Loess Plateau in China covers an area of approximately 640,000 km^2^ and has the largest and thickest loess deposits in the world. Unfortunately, its fragile ecosystems are still challenged by soil erosion and changes in vegetation and land use (Tang, Zhang & Lei, 1998; Shi & Shao, 2000; Zhu et al., 2019). It is worth noting that serious soil and water losses (Shi & Shao, 2000), with the rate of soil loss exceeding 50 t ha ^−^^1^ year ^−^^1^ (Jiao et al., 2008), are associated with the cultivated slopes, which account for approximately 70% of the arable land in the loess hilly and gully areas (Tang, Zhang & Lei, 1998). To restore vegetation and reduce soil erosion on the Loess Plateau, the Chinese government has taken several policies and measures (Jiao et al., 2008; Fu et al., 2011), including the Grain for Green Program across the country to convert massive croplands into abandoned agricultural lands to reduce water loss and soil erosion (Zhang et al., 2013; Li, Qian & Wu, 2014).

After cropland abandonment, the first effect was the start of a secondary succession of plant colonization (Pugnaire et al., 2006) followed by alterations in the soil properties (Awiti, Walsh & Kinyamario, 2008; Cramera, Hobbs & Standisha, 2008). Clements’s models (1928; 1936) indicate that plant succession in abandoned fields should theoretically follow a linear progression, starting from herbaceous plants that give way to shrubs and then trees. In practice, many biotic, abiotic, and anthropogenic factors may interfere with this process and give rise to a wide variety of revegetation patterns, ultimately affecting soil quality (Ruskule, Nikodemus & Kasparinskis, 2016; Peña Angulo et al., 2019; Lasanta, Arnáez & Nadal-Romeroa, 2019; Vahdati et al., 2017). In a way, abandoned croplands provide unique opportunities to understand vegetation succession patterns and how they relate to environmental factors (Cramera, Hobbs & Standisha, 2008; Jiao et al., 2008).

Appropriate multivariate analysis methods, including two-way indicator species analysis (TWINSPAN) (Jafari et al., 2004; Zhang, 2005; Jiao et al., 2007; Jiao et al., 2008; Naqinezhad, Hamzeh’ee & Attar, 2008; Jia et al., 2011; Kargar-Chigani et al., 2017), principal component analysis (PCA) (Naqinezhad, Hamzeh’ee & Attar, 2008; Ruskule, Nikodemus & Kasparinskis, 2016; Kargar-Chigani et al., 2017), detrended correspondence analysis (DCA) (Zhang, 2005; Jiao et al., 2008; Jia et al., 2011; Ruskule, Nikodemus & Kasparinskis, 2016; Kargar-Chigani et al., 2017), canonical correspondence analysis (CCA) (Naqinezhad, Hamzeh’ee & Attar, 2008; Kargar-Chigani et al., 2017) and classification tree model (Jiao et al., 2008; Jia et al., 2011; Wang et al., 2018) can play important roles in identifying the vegetation succession types and their important influencing factors based on quantification of the relationship between the vegetation and environmental factors. On the Loess Plateau of China, several workers investigated the vegetation-environmental relationship by using TWINSPAN, DCA and/or classification tree model (Zhang, 2005; Jiao et al., 2007; Jiao et al., 2008; Jia et al., 2011). These analyses provided useful vegetation succession and soil information, including the succession series of plant communities and soil thresholds as references for guiding vegetation restoration in less than or equal to 50 years. Furthermore, the main vegetation types after cropland abandonment on the Loess Plateau in the short to medium term were *Artemisia scoparia Waldst. et Kit.*, *B. ischaemum*, *L. davurica.*, *Ostryopsis davidiana Decne* and *S. viciifolia* (Zhang, 2005; Jiao et al., 2007; Jiao et al., 2008; Jia et al., 2011). To our knowledge, there is no reported study specifically on the identification of vegetation types and their important influencing factors as well as the thresholds of the important factors for classification of the vegetation types, based on the medium- to long-term succession of natural vegetation after cropland abandonment on the Loess Plateau. We hypothesized that the main vegetation types after cropland abandonment and their important factors in the short to medium term after cropland abandonment on the Loess Plateau should be different from that in the medium to long term. Therefore, we identified the prevalent vegetation types and their important influencing factors as well as the thresholds of the important factors for classification of the vegetation types in the medium-long term after cropland abandonment on the Loess Plateau. Our results would provide insight into natural vegetation succession after cropland abandonment and help make site-specific management for vegetation restoration and the reduction of soil erosion on the mountains and the Loess Plateau.

## Materials & Methods

### Study site

The study area was located in the temperate, semi-arid and forest-steppe zone in the hilly-gullied Loess Plateau of China. The study was conducted in two small watersheds (Zhifanggou and Xiannangou; E 109°13′−109°20′, N 36°42 ′−36°46′, respectively). The mean annual temperature of this area is 8.9 °C with a mean annual precipitation of 535 mm with 75% of the precipitation falling during the flood season from June to September. The soils have developed on the wind-deposited loess parent material and are classified as Calcic Cambisol (Wang et al., 2003). The clay, silt, and sand contents of the soil are 11.7%, 23.7%, and 64.6%, respectively (Wang, Liu & Zhang, 2009).

### Data collection

All quantitative data were obtained in August 2005 and September 2006 using the method by Jia et al. (2011). Under the support of the Ansai Research Station of Soil and Water Conservation, Chinese Academy of Sciences, 42 vegetation plots were selected along the transects and the sampling plots were determined by the community size: the quadrant was 1 m ×1 m in the grasslands (27 plots), 5 m ×5 m in the shrublands (10 plots), and 20 m ×20 m in the woodlands (5 plots). The cover, height, and frequency of each plant species were collected in each quadrant. We obtained information on the length of time (Year) that the croplands had been abandoned from the local landowners and expert knowledge. Soil samples were collected from each plot at six points, 0–40 cm deep, in an S-shaped pattern. The samples from each plot were mixed to reduce soil heterogeneity. There was no rainfall during soil and plant sampling. After that, the soil samples were air-dried and passed through 1-mm and 0.25-mm sieves, respectively. The soil physiochemical properties were measured and analyzed according to Lu (2000). The < 0.001 mm clay percentage (<0.001Clay) was determined using the hydrometer method. The soil water content (WC) at depths of 0–200 cm (SWC0-200) and 200–500 cm (SWC200-500) was measured by oven-drying the samples at 110 °C for 10 h. Water-stable aggregates (WSA) were determined using the Yavinov method. The mean weight diameter (MWD) was obtained using the Van Bavel method. Organic matter (OM) content was determined by K_2_Cr_2_O_7_. The available nitrogen (AN) of the soil was determined using a micro-diffusion technique after alkaline hydrolysis. The available phosphorus (AP) of the soil was determined using the Olsen method. The available potassium (AK) of the soil was measured using flame photometry. The quantity of soil microbes (SM) was analyzed using the dilution plate method (Xu & Zheng, 1986). A life form spectrum was assigned to all vascular plants identified according to the definitions of Raunkiaer (Raunkiaer, 1934).

### Data analysis

The floristic data were quantified by the TWINSPAN to identify the main vegetation types present in the study area (Hill, 1979). Six cut levels were adopted according to the following standards: (1) 0.1%–4%, (2) 4.1%–10%, (3) 10.1%–25%, (4) 25.1%–33%, (5) 33.1%–50%, and (6) 50.1% or greater (Rodwell, 1991).

The program Canoco 4.5 for Windows (Ter Braak & Smilauer, 2002) was used to identify the variables that best discriminated the vegetation types. First, PCA was used to find a general pattern in the measured environmental variables. DCA was then applied to search for major gradients in the composition of species. And CCA was used to analyze the relative importance of the first and second major gradients of environmental variables in explaining the species distribution patterns (Lepš & Šmilauer, 2003). Twelve controlled environmental factors, including soil variables, were involved in our analysis. We tested the effect of all separate variables and whole variables on species composition by using a Monte Carlo permutation test with 999 permutations and a significance threshold of *p* = 0.05 based on the CCA. Manual forward selection was used to identify the subset of factors that could best discriminate among the different vegetation types. Finally, a classification tree model was used to define the critical thresholds of the identified variables determining the vegetation types (Breiman et al., 1984). J48 was adopted as the classification tree model based on Weka 3.8.4 Software (Dale & Reutemann, 2008). The classification tree model was tested by a 10-fold cross-validation, and the model performance was assessed using the kappa statistics.

Univariate analysis was applied using SPSS 17.0. The normal distribution of measured variables was confirmed by the Kolmogorov–Smirnov test. The Pearson correlation coefficient was applied to test relationships between variable values.

## Results

### Main trends in the measured environmental variables

**Figure 1:**
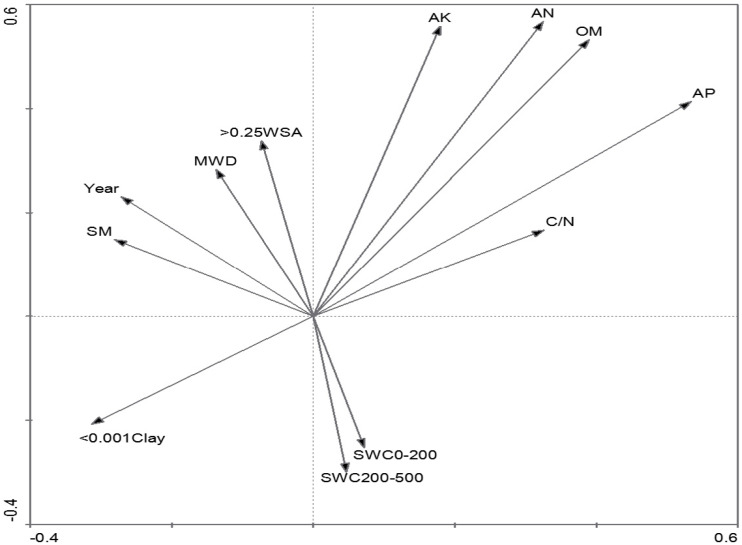
PCA ordination of the investigated environmental variables.

The measured variables were classified into two groups according to the results of PCA, including soil nutrient-related variables and soil texture-related variables (Table 1). The environmental variables on the first axis of PCA were preliminarily classified as soil nutrient-related variables, such as AP and AK (Fig. 1, Table 1). The axis also included the < 0.001Clay, with obviously negative relation with soil nutrient-related variables. The second axis was associated with soil texture-related variables, including soil texture variables (>0.25 WSA and MWD) and water content (Fig. 1, Table 1). The Year was strongly positively correlated with SM, MWD and > 0.25 WSA. SWC was classified as a soil texture-related variable and was negatively correlated with soil texture.

**Table 1:** Descriptive statistics for the investigated variables and the classification of variables into groups (related variables) as well as correlations between the environmental matrix and the first two axes based on the PCA ordination.

	Min	Max	Mean	SD	PCA (axis 1)	PCA (axis 2)	Related variable
Organic matter (OM)	4.40	34.17	10.33	5.93	0.3069	0.0222	Nutrient
C/N	6.07	21.34	13.92	2.44	0.1194	−0.3412	Nutrient
available nitrogen (AN)	16.72	135.10	43.02	24.68	0.3014	0.0387	Nutrient
available phosphorus (AP)	1.68	5.72	2.60	0.76	0.6426	0.2020	Nutrient
available potassium (AK)	62.98	185.80	99.71	24.70	0.3296	−0.1282	Nutrient
<0.001 mm Clay (<0.001Clay)	0.11	3.26	2.56	0.53	−0.4827	−0.2191	Nutrient
>0.25 mm water-stable aggregates (>0.25WSA)	29.80	80.10	53.69	13.30	−0.4513	0.4679	Texture
Mean weight diameter (MWD)	1.01	3.46	2.18	0.63	−0.4874	0.4454	Texture
Soil water content in 0–200 cm (SWC0-200)	3.76	16.49	10.83	3.25	0.3520	0.5473	Texture
Soil water content in 0–500 cm (SWC200-500)	3.95	14.60	10.14	2.89	0.3244	0.4953	Texture
Years since abandonment(Year)	1.00	60.00	23.74	16.52	−0.2712	0.0099	Texture
Quantity of soil microbes (SM)	12.73	77.03	44.71	13.44	−0.1783	0.2510	Texture

### Identification of vegetation types

**Figure 2:**
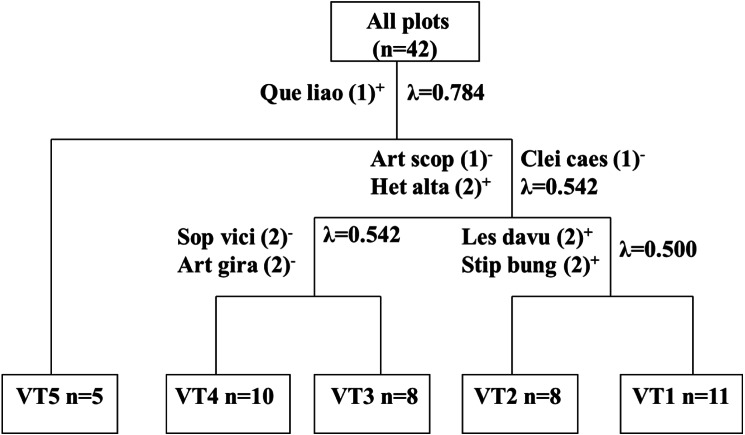
Dendrogram of two way indicator species analysis (TWINSPAN) classification of vegetation on abandonment cropland. n = number of plots; “+” stands for positive indicator species and “-” for negative indicator species; the number in parentheses stands for pseudospecies cut level.

As shown in Fig. 2, five vegetation types were identified by the TWINSPAN cluster analysis. The first division of the clustering dendrogram generated an arbor type of vegetation (VT5) by the indicator species *Quercus liaotungensis*. The second division generated the herb and shrub groups. These two groups were generated by the first level of division in TWINSPAN and were characterized by the indicator species *Artemisia scoparia*, *Heteropappus altaicus,* and *Cleistogenes caespitosa*. The vegetation type was then generated by the third division in the dendrogram and by the second level of TWINSPAN by the indicator species *Sophora viciifolia* and *Artemisia giraldii Pamp* (VT4 and VT3). The third division of the dendrogram generated VT1 and VT2, which were also generated by the second level of division in TWINSPAN, and were characterized by the indicator species *Lespedeza davurica* and *Stipa bungeana*.

VT1 (11 sample plots) was characterized by *Artemisia scoparia* and the presence of some preferential species, including *Heteropappus altaicus*, *Leymus scalenus,* and *Setaria viridis,* which differentiated this group from the rest. VT2 (8 sample plots) was characterized by the indicator species *Lespedeza davurica* and *Stipa bungeana*. This type differed from the rest by the presence of some endemic species, including *Bothriochloa ischaemun*, *Lespedeza davurica*, and *Erudium stephanianum*. VT3 (8 sample plots) was characterized by *Artemisia giraldii pamp* as the dominant and indicator species. This group was unique due to its definitive floristic composition, and *Artemisia giraldii Pamp*, *Artemisia gmelinii*, *Spiraea trilobata,* and *Lespedeza juncea* were the most important preferential species of this type. VT4 (10 sample plots) was characterized by *Sophora viciifolia* as the dominant and indicator species. The presence of *Sophora viciifolia* and some small-shrub species differentiated this type from the others. VT5 (5 sample plots) was characterized by the presence of *Quercus liaotungensis* and *Biota orientalis*, was mostly confined to the forest-zone and was considered to be the final successional stage.

### Major gradients in the vegetation types

**Figure 3:**
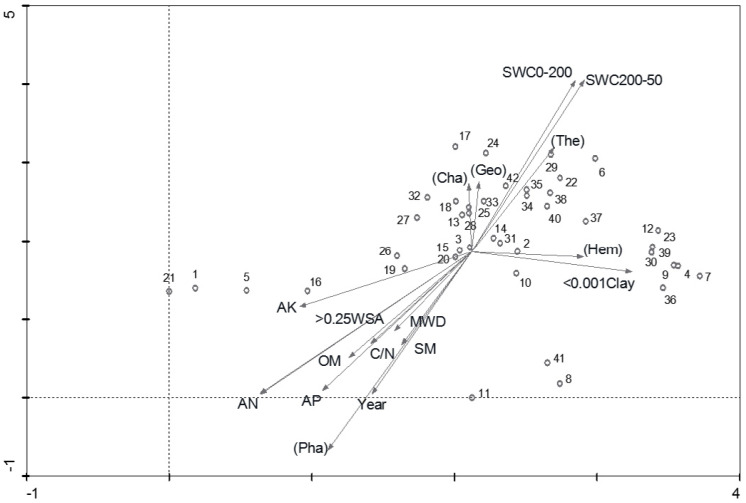
DCA biplot of sampling plots, life forms and environmental variables. Life forms in bracket (Cha =Chamaeophyte, Geo = Geophyte, The = therophyte, Hem = Hemicryptophyte, Pha = Phanerophyte).

The results of DCA presented two major gradients in species data (Fig. 3). The gradient from arbor (VT5) toward shrub was closely related to the first axis. This species structure was significantly correlated with the soil nutrient-related variables. The water-retaining capacity increased on the first axis of DCA from left to right, corresponding with a decrease of soil nutrient content. The second gradient dealing with the separation of other types was related to soil texture.

The vegetation types classified by TWINSPAN were clearly shown in the DCA ordination (Fig. 3). VT1 indicated a high proportion of hemicryptophytes on the first axis. VT2 and VT3 demonstrated a high proportion of geophytes and chamaephytes on the second axis. VT2 also had a high proportion of therophytes, while Phaenerophytes were found in both VT4 and VT5.

**Figure 4:**
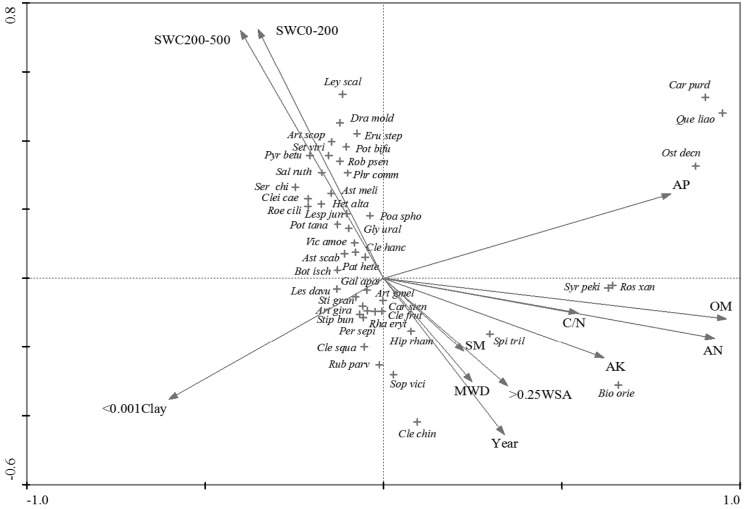
CCA triplot of species and environmental variables. Abbreviated species of diagram: *Artemisia gmelinii, Artemisia giraldii Pamp, Artemisia scoparia, Astragalus melitoloides, Astragalus scaberrimus, Biota orientalis, Bothriochloa ischaemun, Caragana purdomii, Carex stenophylla, Cleistogenes caespitosa, Cleistogenes chinensis, Cleistogenes hancei Keng, Cleistogenes squarrosa, Clematis fruticosa, Dracocephalum moldavica, Erudium stephanianum, Galium aparina, Glycyrrhiza uralensis, Lespedeza davurica, Lespedeza juncea, Leymus scalinus, Heteropappus altaicus, Hippophae rhamnoides, Ostryopsis Decne, Patrinia heterophylla, Periploca sepium, Poa sphondylodes, Potentilla bifurca, Potentilla tanacetifolia, Pyrus betulaefolia, Quercus liaotungensis, Rhamnus erythroxylon, Robinia psendoacacia, Roegneria ciliaris, Rosa xanthina, Rubus parvifolius, Salsola ruthenica, Serratula chinensis, Setaria viridis, Sophora viciifolia, Spiraea trilobata, Stipa bungeana, Stipa grandis, Syringa pekinensis.*

In terms of CCA, the eigenvalues of its first two axes (Fig. 4) were 0.811 and 0.497, respectively. The investigated environmental variables explained 74.3% of total inertia in species data, of which 30.2% could be explained by the first two axes. Furthermore, the first canonical axis was significantly correlated with OM, AN, AP, and AK, followed by < 0.001Clay and C/N, while the second canonical axis was related to SWC and Year (Fig. 4). As for the vegetation types, VT1 and VT2 which correlated with soil water content, were positioned in the west of the CCA diagram. VT5 was separated in the east of the CCA diagram and was related to soil AP, OM, AN, AK and C/N. For the rest types, VT3 and VT4 were positioned in the center of the CCA diagram. It was observed that VT4 was related to the Year, MWD, SM, and > 0.25 WSA while VT3 was related to < 0.001Clay.

### The role of explanatory factors

The effects of environmental variables on vegetation variation based on the manual forward selection are summarized in Table 2. The Year had the highest explanatory power in vegetation variation, followed by OM, AN, and AP as significant soil nutrient factors. As for all other variables but MWD, including < 0.001Clay, SWC200-500, SWC0-200, AK, C/N, SM, and > 0.25 WSA also significantly contributed to the vegetation variation.

**Table 2:** Effect of different environmental variables on the explanation of vegetation variation and their statistical significance.

Variable	Explained inertia	*P* value	Variable	Explained inertia	*P* value
Year	0.760	0.001	SWC0-200	0.418	0.001
OM	0.723	0.001	AK	0.401	0.016
AN	0.613	0.001	C/N	0.299	0.041
AP	0.485	0.001	SM	0.295	0.015
<0.001Clay	0.443	0.004	>0.25WSA	0.266	0.028
SWC200-500	0.419	0.004	MWD	0.256	0.051

### Soil thresholds for the vegetation types

**Figure 5:**
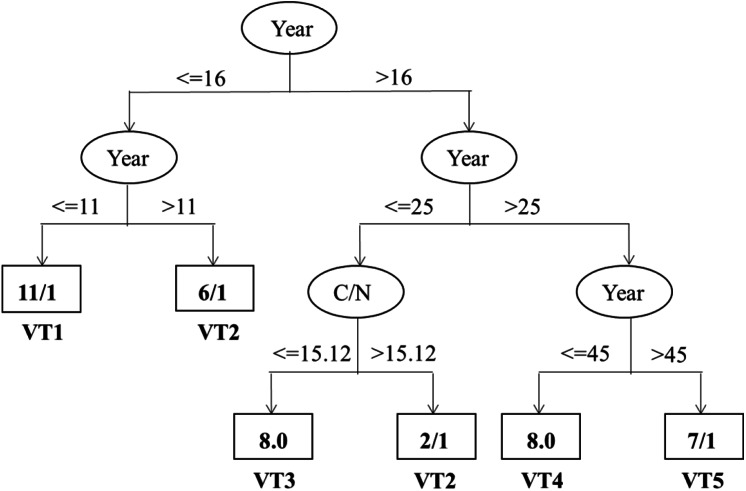
Classification trees discriminating vegetation types and their important influencing factors. The variables and thresholds are shown along the branches.

As shown in Fig. 5, the correct classification proportion and the Kappa statistic were 78.57% and 0.73, respectively. The Year and C/N were found to be the best factors in discriminating vegetation succession types. And the Year showed the maximum information gain ratio and was the first dividing factor. Furthermore, all the five vegetation types were firstly classified based on the 16th year. Based on 11th year, the vegetation types with less than 16 years were then separated into VT1 and VT2. Based on 25th year, the vegetation types with higher than 16 years were then classified into two different mixed types. In the range of 16 years and 25 years, C/N, with its split threshold of 15.12, was the important factor in discriminating between VT2 and VT3. Likewise, the rest mixed types were classified into VT4 and VT5 based on the 45th year when the Year were over 25 years.

## Discussion

The plant communities based on the natural succession of vegetation after cropland abandonment on the Loess Plateau were classified into five vegetation types, including *A. Scoparia*-dominated vegetation (VT1), *L. Davurica-* and *S. Bungeana*-dominated vegetation (VT2), *A. Giraldii*-dominated vegetation (VT3), *S. Viciifolia-* dominated vegetation (VT4), and *Q. Liaotungensis-* and *B. Orientalis*-dominated vegetation (VT5) (Fig. 2). The finding in terms of five vegetation types is consistent with that of Du et al. (2007), Jiao et al. (2007), and Jia et al. (2011) based on the ≤ 50-year abandonment of cropland while the identified vegetation types differentiated. In contrast, the number of vegetation types differs from studies conducted by Jiao et al. (2008) and Zhang (2005)*,* who reported four and seven vegetation types, respectively. These differences may be related to the diverse successional pathways (Naqinezhad, Hamzeh’ee & Attar, 2008) or discrepancies in the abandonment histories (Jia et al., 2011).

Further analysis of the vegetation types showed that the two vegetation types of VT1 and VT5 were the first and the last to appear in the vegetation types, respectively (Table 3, Fig. 3). According to the classification tree (Fig. 5), VT5 with > 45 years of cropland abandonment revealed that the natural recovery of forest vegetation after land abandonment in such arid and semi-arid areas as the Loess Plateau is very slow, especially where water is a key resource in the development of plant cover (Ruiz-Sinoga & Martínez-Murillo, 2009; Jiao et al., 2008).

**Table 3:** Soil variables among different vegetation types in the study area.

Variable	VT1	VT2	VT3	VT4	VT5	All plots
OM (g kg^−1^)	6.78c ±1.12	7.65c ±2.08	9.08c ±2.92	14.51b ±4.20	20.02a ±9.02	11.10 ±6.78
C/N (%)	13.10c ±0.97	13.83bc ±3.44	12.56c ±2.78	15.25ab ±1.63	16.41a ±1.86	14.11 ±2.47
AN (mg kg^−1^)	27.68c ±4.79	27.14c ±9.52	44.12bc ±14.02	58.51b ±27.43	81.34a ±36.22	45.73 ±27.38
AP (mg kg^−1^)	2.77ab ±0.72	2.27b ±0.52	2.30b ±0.37	2.80ab ±0.94	3.34a ±1.24	2.69 ±0.84
AK (mg kg^−1^)	85.44c ±14.57	91.32bc ±18.02	106.96abc ±32.62	108.75ab ±29.24	122.24a ±25.17	101.32 ±26.73
<0.001Clay (%)	2.59a ±0.35	2.70a ±0.32	2.58a ±0.44	2.60a ±0.26	2.24a ±1.11	2.56 ±0.51
>0.25WSA (%)	48.18b ±13.62	47.19b ±16.25	54.95ab ±9.41	60.24a ±8.64	65.35a ±11.19	54.63 ±13.26
MWD (mm)	1.95c ±0.71	1.67bc ±0.40	2.35ab ±0.38	2.51a ±0.37	2.69a ±0.69	2.22 ±0.62
SWC0-200 (%)	13.66a ±1.64	11.43ab ±1.77	10.96b ±3.47	8.31c ±2.49	7.30c ±2.18	10.59 ±3.26
SWC200-500 (%)	12.65a ±1.31	11.08ab ±1.67	10.09b ±2.95	7.92c ±2.07	6.65c ±1.73	9.92 ±2.90
Year	7.64e ±4.03	15.29d ±2.93	21.88c ±2.36	35.00b ±7.82	54.17a ±4.92	24.79 ±16.44
SM (CFU)	33.94b ±4.06	37.80b ±4.53	42.93b ±7.79	39.73b ±19.61	68.12a ±7.03	42.56 ±15.14

**Notes.**

Different letters mean in the same row are significantly different at *p* = 0.05.

As for VT1, the results are similar to Jia et al. (2011) who found annual or biennial species (e.g., *A. scoparia*) were dominant in the early stages. The other three vegetation types were observed with rather different sets of the floristic structure (Fig. 3), although there are habitats similarities among them. The high proportion of therophytes in VT2 was probably due to the decline in soil water compared with VT1 (Fig. 4) and encroachment of some annual weeds after a temporary human destruction (Jiao et al., 2008; Jia et al., 2011). By contrast, the geophytes dominated in VT3 with the chamaephyte in VT4 (Fig. 3), indicating that the two vegetation types (VT3 and VT4) showed substantial structural and floristic heterogeneity. This may be due to the effects of environmental variables, such as Year, MWD, SM and >0.25 WSA for VT4, < 0.001 clay for VT 3 (Fig. 4), which might control the separation of different vegetation communities on the hilly-gullied Loess Plateau, since the vegetation succession patterns in our study share strong homogeneity in both topography and climatic factors (Zhou, Shanguan & Zhao, 2006).

Since the classification and ordination methods could generate results that were complementary and ecologically meaningful (Naqinezhad, Hamzeh’ee & Attar, 2008), DCA and CCA were adopted to explain the power of the investigated environmental variables in the inertia in species data. Interestingly, they showed similar patterns in vegetation variation for environmental variables along the axes (Figs. 1, 3 and 4). Furthermore, the first axis separated soil nutrient variables, which were very important factors in affecting the separation of plant communities (Jiao et al., 2008; Vahdati et al., 2017). As for the soil texture-related variables, the effects of < 0.001 Clay and > 0.25 WSA on the first axis of DCA and CCA were more than the effects of the two variables on the first axis of PCA, while SWC is on the opposite (Figs. 3 and 4). Compared with the first axis, the < 0.001 Clay and > 0.25 WSA were observed with major effects on the vegetation grouping on the second axis (Fig. 4). Many studies (Jia et al., 2011; Adel, Pourbabaei & Dey, 2014; Zhang et al., 2018) have shown that clay content could be accompanied by accumulation of soil organic and mineral texture, probably finally affecting the vegetation grouping together. In addition, no significant differences in < 0.001 Clay and > 0.25 WSA among five succession types were observed, respectively (Table 3). As for SWC, a significant difference among five succession types was observed (Table 3), with a major effect on the vegetation grouping on the second axis compared with the first axis (Fig. 4). In sum, we could conclude that both soil nutrient variables and soil texture-related variables directly affected the vegetation grouping, with a stronger role of soil nutrient variables compared with soil texture-related variables.

Based on the manual forward selection of CCA, the results indicated that all variables except MWD had a significant effect on vegetation variation (Table 2). In particular, the Year was identified as the most important factor influencing the vegetation variation (Table 2), as also indicated by the Fig. 5. Our finding is qualitatively in-line with the results of a number of studies showing that the succession process of plant communities is mainly influenced by the period of time since cropland abandonment (Zhang, 2005; Du et al., 2007; Jiao et al., 2007; Jiao et al., 2008; Jia et al., 2011; Lasanta, Arnáez & Nadal-Romeroa, 2019). The explained inertia of 0.760 by the Year is higher than that of 0.53 reported by Jiao et al. (2008), who also identified the factors affecting the process of vegetation succession in the forest-steppe region on the Loess Plateau. Reportedly, soil quality is frequently considered to be an important factor in determining vegetation succession (Lasanta, Arnáez & Nadal-Romeroa, 2019). In this study, OM was identified as an important soil factor with the explained inertia value of 0.723. Calsamiglia et al. (2017) found that soils in recently abandoned croplands had little organic content. When land abandonment is followed by vegetation succession, it generally helps to improve soil quality, including OM (Jia et al., 2005; Tasser et al., 2007; Deng et al., 2017; Liu et al., 2020). Our study was conducted on the basis of longer natural vegetation succession after cropland abandonment, probably resulting in higher amounts of organic matter in the soil from the conversion from C_4_ vegetation to C_3_ vegetation with an increase in abandonment years (Deng et al., 2017; Deng et al., 2018), as also indicated by the finding that VT5 was related to soil OM (Figs. 3, 4). Generally, nitrogen, phosphorus and potassium are essential elements for plant growth and act as determinants of biomass, plant species composition, and plant replacement (Jiao et al., 2008). Soil AN, AP and AK were also identified as important factors influencing vegetation variation, as partially supported by that VT5 was related to soil AP, AN and AK (Figs. 3 and 4), which is consistent with previous studies (Jiao et al., 2008; Jia et al., 2011).

As for soil-texture variables, the soil water content in the 0–200 cm and 200–500 cm soil layers was also found to be important factor on vegetation variation (Table 2), as also indicated by the significant relationship between soil water content as well as VT1 and VT2 (Fig. 4). Reportedly, soil moisture is a key factor in plant distribution and growth, and water scarcity is frequently considered to be a limiting factor in affecting vegetation succession types after cropland abandonment on the Loess Plateau (Jiao et al., 2008; Jia et al., 2011). The mean soil water content of different vegetation types was approximately 10% (Table 1), which is nearly half of the field water capacity (Jiao et al., 2008), suggesting that the mean soil water status is severely deficient for vegetation restoration to take place. Likewise, the soil water content in different soil layers was significantly negatively related to the Year and most of the investigated soil properties (Table 4, Fig. 4). These findings are consistent with the results of Zhang et al. (2016) in which soil water content significantly decreased along the selected succession stages of vegetation from the early grass stage to the climax forest stage. However, these results differ from those in which soil water content increased with vegetation succession in China’s rocky Qinling Mountains (Zhang et al., 2019). The decreasing trend of soil water content in the surface soil with the vegetation succession (Table 3) should be emphasized when planning or managing the vegetation and soil in the abandoned croplands on the Loess Plateau for the medium-long term.

**Table 4:** Correlations between the investigated environmental variables based on the vegetation communities after cropland abandonment on the Loess Plateau.

	OM	C/N	AN	AP	AK	>0.25WSA	MWD	SWC0-200	SWC200-500	Year	<0.001Clay
C/N	0.685**										
AN	0.975**	0.571**									
AP	0.688**	0.368*	0.674**								
AK	0.717**	0.436**	0.788**	0.560**							
>0.25WSA	0.469**	0.269*	0.464**	0.265*	0.339*						
MWD	0.377*	0.208	0.394**	0.192	0.252	0.908**					
WC0-200	−0.522**	−0.497**	−0.525**	−0.151	−0.422**	−0.416**	−0.346*				
WC200-500	−0.571**	−0.528**	−0.583**	−0.204	−0.516**	−0.471**	−0.407**	0.970**			
Year	0.710**	0.501**	0.696**	0.246	0.417*	0.511**	0.521**	−0.642**	−0.681**		
<0.001Clay	−0.479**	−0.204	−0.496**	−0.357*	−0.396**	−0.143	−0.163	0.115	0.161	−0.276	
SM	0.242	0.141	0.260*	−0.069	0.095	0.206	0.274	−0.115	−0.152	0.653**	−0.243

Compared with the > 0.25 WSA under herbs, our results agree with earlier studies in which > 0.25 WSA were more stable under different succession stages, especially late succession stages (Zhu, Li & Li, 2010). Actually, > 0.25 WSA is frequently considered to be an important factor influencing vegetation variation and soil aggregate stability which was improved as secondary succession took place (Duchicela et al., 2013; Erktan et al., 2016). Although MWD is another important associated variable of > 0.25 WSA (Table 4), it was not identified as an important factor influencing vegetation variation. This may be due to the difficulty in improving the physical conditions of the soil, including MWD and < 0.001Clay, in the short- to medium-term natural recovery period (Zhang et al., 2019; Zhai et al., 2020). As an important factor influencing vegetation variation, < 0.001Clay nominally decreased with increasing years after cropland abandonment, which differs from the results of Pernes-Debuyser et al. (2003), Zhang et al. (2016), Zhang et al. (2018), probably due to the different vegetation types and years of abandonment. Considering that the microbial composition and diversity of the soil may change concomitantly with successive vegetation stages (Jia et al., 2005; Zhang, 2005; Jia et al., 2011; Cline & Zak, 2015), SM could be an important factor in determining the vegetation types based on the natural vegetation succession after cropland abandonment on the Loess Plateau (Table 2).

Based on the classification tree model, further analysis indicates that Year and C/N were found to be the best factors for discriminating vegetation succession types (Fig. 5). In particular, only the two factors existed in the classification tree model, probably owing that the Year was significantly related to most of the investigated variables (Table 4). Consequently, the other variables may be wholly or partially displaced by the Year when identifying the most important factors influencing vegetation succession types. These results further highlight the important role of time after cropland abandonment in determining the vegetation types. In a way, the years after cropland abandonment may be considered as a prerequisite indicator to denote the typical vegetation types. Previously, the reported studies had a shorter period of less than or equal to 50 years, which is less than that of the present study (60 years) (Zhang, 2005; Du et al., 2007; Jiao et al., 2007; Jiao et al., 2008; Lasanta, Arnáez & Nadal-Romeroa, 2019). Our study could provide supporting evidences for classification of the vegetation types and identification of their important factors in a more robust way. Likewise, C/N was also found to be an important factor discriminating vegetation types, which is different from Jia et al. (2011) who identified AN, soil water, and TP as the best discriminating factors among vegetation types. It is worth noting that the threshold of C/N with a value of 15.12 for discriminating between the VT2 (*L. Davurica-* and *S. Bungeana*) and VT3 (*A. Giraldii*) was within the range of Zhang et al. (2012) on the Loess Plateau. The relative high threshold value of C/N probably resulted from asynchronous accumulation of the SOC and total N, with a faster accumulation rate of SOC than that of total N during the early vegetation succession (Tong et al., 2009; Xue & An, 2018; Liu et al., 2020). Paniagua et al. (1999) have shown that soil properties change with the process of vegetation succession and soil conditions in a certain successional stage may reflect their suitability for the establishment of the plant species during the same stage (Paniagua et al., 1999; Jia et al., 2011). From the perspective of speeding up ecological restoration, the site-specific introduction of typical vegetation types at given sites may allow to efficiently restore vegetation and control soil erosion of the region based on quantification of the environmental factors, especially the time after cropland abandonment and soil C/N, considering that a long time interval is required to proceed natural vegetation succession to obtain target vegetation types after cropland abandonment and such timeframe is difficult to accept in this region due to its very rapid and extensive soil erosions (Zhao et al., 2005; Jiao et al., 2008; Jia et al., 2011).

Our study explored the successive natural vegetation and its important influencing factors after cropland abandonment on the Loess Plateau. Zhang et al. (2019) showed the effect of a century of vegetation gradients following agricultural abandonment on edaphic characteristics, soil microbial community, C and N mineralization rates, and functional genes. Although our study was conducted based on the natural vegetation with the longest 60-year-old forest communities, additional empirical studies and longer investigations into the types of vegetation succession are needed to clarify the typical vegetation types based on the successive vegetation after cropland abandonment on the Loess Plateau, since natural vegetation recovery in a semi-arid environment is slow and follows multiple routes of succession after land abandonment (Lasanta, Arnáez & Nadal-Romeroa, 2019; Liu et al., 2020).

## Conclusion

Our results indicated that all of the investigated vegetative communities with longest 60-year-old forest communities could be classified into five distinctive vegetation types based on the investigation of natural vegetation succession after cropland abandonment on the Loess Plateau. It was observed that soil nutrient variables showed a stronger role in shaping the vegetation grouping compared with soil texture-related variables. Furthermore, Year and C/N were identified as the best factors for discriminating vegetation succession types. These results provide supporting evidence for the hypothesis that the main vegetation types after cropland abandonment and their important factors in the short to medium term after cropland abandonment on the Loess Plateau differentiated from that in the medium to long term. Likewise, it was observed that most of the investigated soil nutrient variables and soil texture-related variables improved with the vegetation succession. It is worth noting that the soil water in the surface soil layer showed a decreasing trend with a potential risk for vegetation restoration. These information will provide insight into the introduction of typical vegetation types at given sites in a site-specific way to speed up restoring suitable vegetation types and controlling soil erosion after cropland abandonment on the Loess Plateau. Our results encourage further exploration of vegetation succession and their important factors based on longer periods of vegetation succession after cropland abandonments under more soil and climatic conditions on the mountainous areas as the Loess Plateau.

##  Supplemental Information

10.7717/peerj.10349/supp-1 Supplemental Information 1 Raw data

## Additional Information and Declarations

### Field Study Permissions

The following information was supplied relating to field study approvals (i.e., approving body and any reference numbers): Field experiments were approved by the Ansai Research Station of Soil and Water Conservation, Chinese Academy of Sciences.

### Data Availability

The following information was supplied regarding data availability: The raw measurements are available as a Supplemental File.
